# Structure and Properties of Fe–Al–Si Alloy Prepared by Mechanical Alloying

**DOI:** 10.3390/ma12152463

**Published:** 2019-08-02

**Authors:** Pavel Novák, Tomáš Vanka, Kateřina Nová, Jan Stoulil, Filip Průša, Jaromír Kopeček, Petr Haušild, František Laufek

**Affiliations:** 1 Department of Metals and Corrosion Engineering, University of Chemistry and Technology, Prague, Technická 5, Prague 6, 166 28 Prague, Czech Republic; 2 Institute of Physics of the ASCR, v. v. i., Na Slovance 2, Prague 8, 182 21 Prague, Czech Republic; 3 Department of Materials, Faculty of Nuclear Sciences and Physical Engineering, Czech Technical University in Prague, Trojanova 13, Prague 2, 120 00 Prague, Czech Republic; 4 Czech Geological Survey, Geologická 6, Prague 5, 152 00 Prague, Czech Republic

**Keywords:** iron silicide, Fe–Al–Si alloy, mechanical alloying, spark plasma sintering, characterization

## Abstract

Fe–Al–Si alloys have been previously reported as an interesting alternative to common high-temperature materials. This work aimed to improve the properties of FeAl20Si20 alloy (in wt.%) by the application of powder metallurgy process consisting of ultrahigh-energy mechanical alloying and spark plasma sintering. The material consisted of Fe_3_Si, FeSi, and Fe_3_Al_2_Si_3_ phases. It was found that the alloy exhibits an anomalous behaviour of yield strength and ultimate compressive strength around 500 °C, reaching approximately 1100 and 1500 MPa, respectively. The results also demonstrated exceptional wear resistance, oxidation resistance, and corrosion resistance in water-based electrolytes. The tested manufacturing process enabled the fracture toughness to be increased ca. 10 times compared to the cast alloy of the same composition. Due to its unique properties, the material could be applicable in the automotive industry for the manufacture of exhaust valves, for wear parts, and probably as a material for selected aggressive chemical environments.

## 1. Introduction

Materials based on Fe–Al ordered phases have been subjected to extensive research and development over the last decades, due to their low cost, low density, high specific strength, high creep resistance, as well as excellent high-temperature oxidation and sulphidation resistance [1]. The positive effect of aluminium on the heat-resistance of iron-based alloys was reported already in 1894 [2]. However, because of room temperature brittleness and manufacturing problems, these alloys were only sporadically applied for a long time. A significant advance in the production of these materials was achieved in former Czechoslovakia in the 1950s, where a Fe–Al–C alloy called Pyroferal was developed [3], being composed of FeAl phase and particles of Al_4_C_3_ carbide [3]. This material with excellent oxidation resistance and good high-temperature mechanical properties was designed to be produced by casting, which made it easily producible and cost-efficient [3]. However, several problems arose during application of this material. In acidic solutions or high-temperature environments with water vapour, aluminium carbide was hydrolysed to methane, which destroyed the material. Therefore, the extensive search for adequate carbon-free Fe–Al alloys began. Several alloy types containing Nb, Ta, Cr and other elements have been developed [4,5,6,7,8,9,10]. However, due to the economic situation of the EU, most of these elements are now listed as critical raw materials (CRMs) [11]. Hence, the development of these alloys is not preferred. 

Recently, Fe–Al–Si alloys have been developed by our team as another carbon-free alternative to iron-based intermetallic alloys. Even though silicon is also listed as a CRM, the problem can be easily solved by the use of silicon recovered from recycled electronics, because a lower purity is required for these alloys than that used in electronics. These alloys are characterised by extremely good high-temperature oxidation resistance in air and hardness [12]. The oxidation resistance was found to be given by aluminium oxide layer on the surface and also additionally by the sub-layer enriched by iron silicides as a result of the consumption of aluminium upon formation of the oxide layer [12]. Further published papers also demonstrated the exceptional oxidation resistance of Fe–Si–Al ternary alloy in CO_2_–H_2_O gaseous atmospheres [13]. Since the processing of iron-based intermetallics by conventional metallurgical routes is relatively problematic [14,15,16], powder metallurgy using reactive sintering has previously been tested for the Fe–Al–Si alloys [17]. Even though a deep optimisation of the process was carried out [17], there was still a significant portion of residual porosity which affected the results of the high-temperature oxidation tests, as internal oxidation was observed in the case of some Fe–Al–Si alloys [12]. In addition, the microstructure was relatively coarse, and the alloys were determined to be very brittle at the room temperature, having a fracture toughness of ca. 0.35 MPa·m^1/2^. Due to high residual porosity of the samples produced by reactive sintering, it was not possible to measure the intrinsic mechanical and corrosion properties (e.g., tensile/compressive strength, wear resistance, corrosion behaviour in electrolytes, etc.) and, therefore, they remain unknown for this alloy.

Since it has been reported for several alloy systems that structure refinement could lead to an increase in fracture toughness [18,19] and, also, in order to overcome the abovementioned manufacturing obstacles, a different powder metallurgical route was tested for the preparation of this alloy—a combination of ultrahigh-energy mechanical alloying and spark plasma sintering. Mechanical alloying, as one of the most efficient techniques applicable to achieve fine-grained structure [20,21], is in fact high-energy milling, usually using ball mills. In this process, the high kinetic energy of balls causes the following phenomena: crushing of particles leading to the reduction of the particle size; local mechanical joining and welding of particles by plastic deformation, friction forces and diffusion; structure refinement due to severe plastic deformation; and formation of solid solutions and chemical compounds (intermetallics) [22]. The process of mechanical alloying usually takes tens of hours [23,24,25], which is its main disadvantage. In our previous work [26] we developed the ultrahigh-energy mechanical alloying process, which enables intermetallics to be obtained from metallic powders already in the time between 120 and 240 min. Our process uses high ball-to-powder ratio (over 50:1) and high rotational velocity (at least 400 rpm). No lubrication medium is added in our technology in order to maximise the friction forces between the balls, powder, and wall of the milling vessel [26]. 

Spark plasma sintering (SPS) is a modern compaction method which uses uniaxial pressing accompanied by passage of the electric current through the sample. In the SPS process, rapid heating of the sample is caused by Joule heat, which accompanies the passage of high electric current and electric discharges (spark or plasma) between particles [27,28,29]. The SPS method is especially suitable for the consolidation of nanocrystalline materials and phases with lower thermal stability due to high sintering rate and a corresponding reduction of thermal exposure of the consolidated material [30]. 

In this work, the combination of unique ultrahigh-energy mechanical alloying and spark plasma sintering was tested in order to improve the mechanical properties of FeAl20Si20 alloy (in wt %). This alloy composition has previously been determined to be the most resistant against high-temperature oxidation among Fe–Al–Si alloys [12], but also highly brittle at room temperature when being produced by reactive sintering powder metallurgy or casting. Complex characterisation of the alloy prepared by the abovementioned process was carried out in order to find the future application range for this alloy.

## 2. Materials and Methods

FeAl20Si20 alloy (in wt %) was prepared by the combination of mechanical alloying (MA) and subsequent spark plasma sintering (SPS). Mechanical alloying was carried out in a planetary ball mill (PM 100 CM, Retsch, Haan, Germany)) under the following conditions, as optimised in our previous paper dealing with the synthesis of intermetallics [26]:

- milling duration: 240 min;
- change of rotation direction each 30 min;
- rotation speed: 400 rpm;
- atmosphere: argon;
- powder batch: 5 g;
- ball-to-powder weight ratio: 70:1.

For mechanical alloying, the following elemental powders were applied: iron (purity 99.9%, particle size <44 μm, supplied by Strem Chemicals, Newburyport, MA, USA), aluminium (purity 99.7%, particle size <44 μm, supplied by Strem Chemicals). and silicon (purity 99.5%, particle size <44 μm, supplied by Alfa Aesar, Haverhill, MA, USA). 

The powder was consolidated by SPS method using a HP D10 device (FCT Systeme GmbH, Rauenstein, Germany). A pressure of 48 MPa was applied at 1000 °C for 10 min with a previous heating rate of 300 K/min and a cooling rate of 50 K/min. The weight of the batch for sintering was 5 g. 

The microstructure of the alloys produced by a combination of ultrahigh-energy mechanical alloying and spark plasma sintering was studied using a VEGA 3 LMU scanning electron microscope (TESCAN, Brno, Czech Republic) after etching using modified Kroll’s reagent (5 mL HNO_3_, 10 mL HF, and 85 mL H_2_O). Phase composition was identified by X-ray diffraction analysis (XRD) using a X’Pert Pro X-ray diffractometer (PANalytical, Almelo, The Netherlands). The amounts of phases, and their lattice parameters and crystallite sizes were calculated from the XRD data using Rietveld pattern refinement by the means of Topas 5 software. 

The distribution of the phases in the sample was imaged using an electron backscatter diffraction (EBSD) analyser (manufactured by EDAX) on a FERA III scanning electron microscope (TESCAN, Brno, Czech Republic). Porosity was measured by image analysis (ImageJ 1.48) on the polished non-etched samples as the area fraction of pores. 

Mechanical properties of the SPS-consolidated material were determined by the means of hardness measurement, determination of fracture toughness, and compression tests. Hardness was measured using the Vickers method with a load of 30 kg. Fracture toughness was determined by the indentation test (Vickers indenter FM-700 (Future-Tech, Kawasaki-City, Japan) with the load of 1 kg) and evaluated from the length of cracks by Palmqvist Equation (1) [31]:

(1) KC=0.016×(EHV)12×(Fc32)

where *E* is the Young´s modulus (Pa), *HV* is the Vickers hardness, *F* is the applied load (N), and *c* is the half length of the crack after indentation (m).

Compression tests were carried out using LabTest 5.250SP1-VM universal loading machine (produced by LaborTech, Kateřinky, Czech Republic) at the following temperatures: room temperature, 400, 500, 600, and 700 °C with the initial deformation rate of 0.001 s^−1^. 

The wear resistance was measured using modified pin-on-disc method, where the “pin” was the tested sample and “disc” was a grinding paper P1200. The normal force used in the test was 5.8 N. The wear rate was calculated from the measured weight losses by the Equation (2) [32]:

(2) $$w=\frac{\Delta m\times 1000}{\rho \times l}$$

where *w*, Δ*m*, *ρ*, and *l* are wear rate (mm^3^·m^−1^·N^−1^), weight loss (g), density (5.91 g·cm^−3^), and sliding distance on the grinding paper (2500 m), respectively. The density of the material was determined by the Archimedes method. 

Cyclic and isothermal oxidation tests were carried out at 800 and 1000 °C in air. Oxidation rate was determined from weight gains caused by the oxide formation on the surface of thermally exposed samples. In isothermal oxidation tests, the samples were heated continuously in alumina crucibles for 100, 200, 300, and 400 h, followed by air-cooling. 

Cyclic oxidation tests were applied in order to reveal the susceptibility of the oxide layers to the spallation due to thermally induced stresses. The duration of one oxidation cycle was 48 h. After each cycle, samples were air-cooled, weighed, and heated again to the test temperature.

The microstructure of the oxide layers was documented using a VEGA 3 LMU scanning electron microscope (TESCAN, Brno, Czech Republic), and phase composition was determined by XRD. Glow discharge optical emission spectroscopy (GDOES, Horiba JobinYvon GD Profiler II) was applied for a depth profile chemical analysis of the oxide layers.

To evaluate the oxidation kinetics, the parabolic rate constant was calculated for all oxidation durations according to Equation (3) [33]:

(3) $$k_{p}=\frac{{\left(\frac{\Delta m}{A}\right)}^{2}}{t}$$

where *k_p_*, Δ*m*, *A*, and *t* are parabolic rate constant (g^2^·m^−4^·s^−1^), weight gain (g), exposed area (m^2^), and duration of oxidation (s), respectively. 

The thermal stability of the alloy was tested by the evaluation of microstructure and measurement of Vickers hardness after annealing at 800 and 1000 °C. The hardness was measured in the core of the material on a crosscut.

In order to test the behaviour of the material in water-based electrolytes, the FeAl20Si20 alloy was characterised by means of electrochemical impedance spectroscopy (EIS) on FAS2 potentiostat (Gamry Instruments, Warminster, PA, USA). A disc sample 20 mm in diameter and 5 mm thick was polished by grinding paper P220 prior to each measurement. Samples were exposed in a pressure cell and the O-ring defined the tested area to 0.8 cm^2^. Conditions were settled for 60 min in the testing solution and then the measurement was started. EIS spectra were measured within the range of frequencies from 10 kHz to 1 mHz for less aggressive media and from 10 kHz to 10 mHz in more aggressive media. Sampling was conducted using 5 points per decade and a testing amplitude 20 mV according to open circuit potential (EOC). Saturated silver-silver chloride electrode (ACLE) was used as reference and Pt wire as a counter electrode. 

Study of the material corrosion behaviour was carried out in the series of sulphuric acid solutions with pH 0, 1, 2, 3, 4, and 5.6 (which is demineralised water with dissolved carbon dioxide), in a water-based solution of 0.5 mol·dm^−3^ NaCl and in a solution of 2.2 g·dm^−3^ NaF in demineralised water. X-ray photoelectron spectroscopy (XPS) analysis using ESCAProbeP device (Omicron Nanotechnology, Abingdon, UK) equipped with an Al Kα (λ = 1486.7 eV) X-ray source was used to determine the chemical composition of the passive layer after exposure in a water-based environment. The spectra were measured with an energy step of 0.05 eV and normalised to the binding energy of C1s peak (285.0 eV). Measured spectra were evaluated in CasaXPS 2.3.15 software (IMFP, RSf etc are part of the software library). The data for the chemical state evaluation were obtained from the NIST (Aithersburg, MD, USA) X-ray Photoelectron Spectroscopy Database [34].

## 3. Results

### 3.1. Microstructure and Phase Composition of FeAl20Si20 Alloy

**Figure 1:**
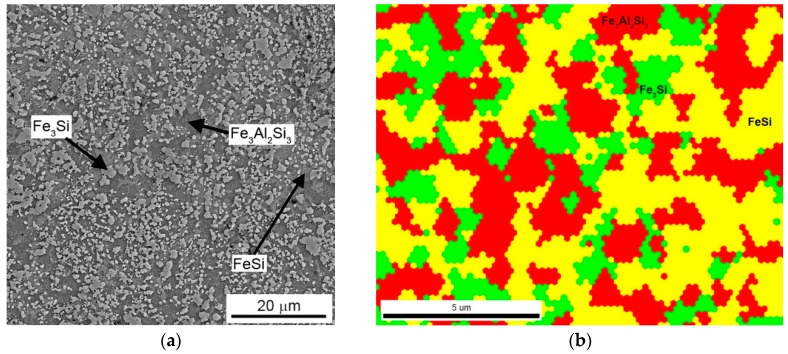
(**a**) Microstructure of FeAl20Si20 alloy produced by mechanical alloying and spark plasma sintering; (**b**) EBSD phase map of the alloy.

**Figure 2:**
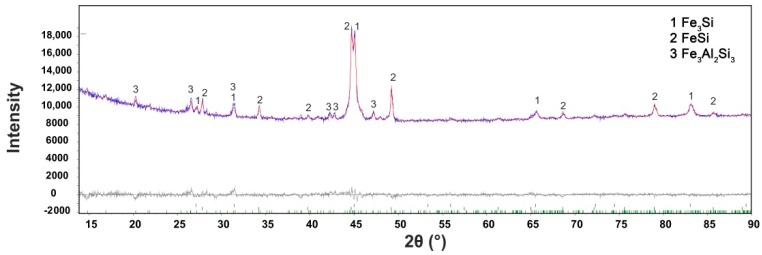
Rietveld plot of FeAl20Si20 alloy produced by mechanical alloying and spark plasma sintering.

Microstructure of the FeAl20Si20 alloy prepared by mechanical alloying and spark plasma sintering is shown in Figure 1a. The alloy exhibits very low porosity (0.1 vol %). The alloy is composed of two types of iron silicides (FeSi and Fe_3_Si) and the Fe_3_Al_2_Si_3_ ternary phase. The distribution of individual microstructure constituents and overall phase composition were characterised by EBSD (Figure 1b), XRD (Figure 2), and EDS (Table 1). 

The amount of Fe_3_Si phase (Fm3m) was determined as 33.0 wt %. The lattice parameter was determined as 5.693 × 10^−10^ m, which is higher than the value presented in the PDF2 database (5.6700 × 10^−10^ m). The same behaviour was observed for FeSi phase (P2_1_3 structure), where the measured value of the lattice parameter reached 4.533 × 10^−10^ m, while the table value was 4.485 × 10^−10^ m. This indicates possible supersaturation of the phase by silicon or partial substitution or enrichment by aluminium. The presence of aluminium dissolved in silicides was proved by the EDS chemical microanalysis (Table 1). In the case of materials prepared by mechanical alloying, this kind of supersaturation is relatively common because the mutual solubility of elements is usually strongly increased by mechanical alloying. This is even observed in the examples, where mechanical alloying produced a solid solution of normally non-miscible elements, such as Mg and Fe [35]. 

The crystallite size of present phases is in the interval of 20–45 nm (Table 2), even though the size of individual particles of silicides reached approximately 0.2–5 μm (Figure 1a). This shows that the silicide particles observed by SEM are, in fact, polycrystals constituted of small nanosized grains. The crystallite size of Fe_3_Al_2_Si_3_ ternary phase is ca. two times higher than this parameter of silicides. The probable reason is in the fact that the ternary phase was not present in the as-milled state [36] and thus formed during sintering, probably by the reaction of silicide phase and iron aluminide (FeAl with B2 structure), which disappeared during the sintering process. The determined phase composition corresponds well qualitatively with the Fe–Al–Si equilibrium phase diagram at 1000 °C [37]. However, a higher ratio between ternary phase and silicides could be expected, because the chemical composition of the alloy is very close to the ternary phase (Table 1).

### 3.2. Mechanical and Tribological Properties of FeAl20Si20 Alloy

The hardness of the alloy reaches 811 ± 19 HV30 and the fracture toughness achieves the value of 3.50 ± 0.33 MPa m^1/2^. The fracture toughness reaches rather low value, comparable mostly with ceramic materials (e.g., corundum-based ceramics) [38]. However, when the same alloy is produced by casting, it exhibits nearly ten times lower fracture toughness (0.35 ± 0.02 MPa m^1/2^). This demonstrates that the structure refinement by mechanical alloying helps to reduce the room temperature brittleness of intermetallics.

The mechanical properties of the FeAl20Si20 alloy are summarised in Table 3. At room temperature, the alloy exhibits yield strength (YS) and ultimate compressive strength (UCS) of 1071 and 1085 MPa, respectively. The temperature dependence of yield strength and UCS shows anomalous behaviour. Both of these mechanical characteristics increase strongly at 500 °C and then rapidly decrease with temperature (Table 3). The abrasive wear rate of the FeAl20Si20 alloy was determined to be 4.5 ± 0.13 mm^3^ m^−1^. For comparison, the AISI D2 cold work tool steel after the recommended regime of heat treatment achieves a value of approximate 15 mm^3^ m^−1^ under the same test conditions. The results show that without the need for any heat treatment, the FeAl20Si20 alloy exhibits more than three times better wear resistance than properly heat-treated cold work tool steel with comparable compressive strength. Due to the absence of heat treatment, it can be expected that the wear resistance would not degrade strongly even when the temperature will increase, as is common during high-speed machining.

### 3.3. High-Temperature Oxidation

**Figure 3:**
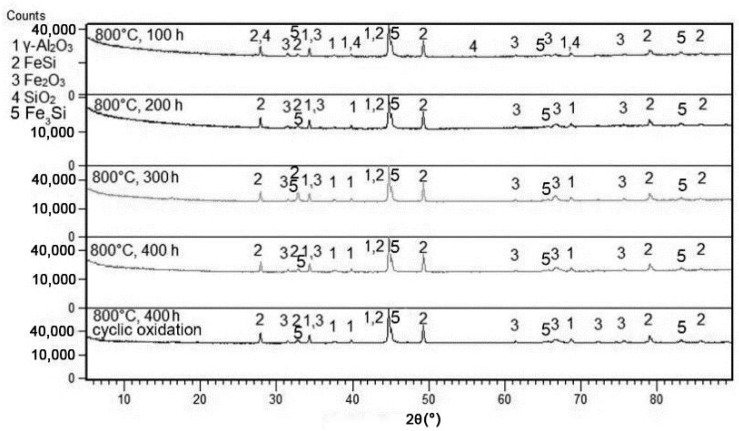
XRD patterns of FeAl20Si20 alloy after various durations of oxidation at 800 °C in air.

**Figure 4:**
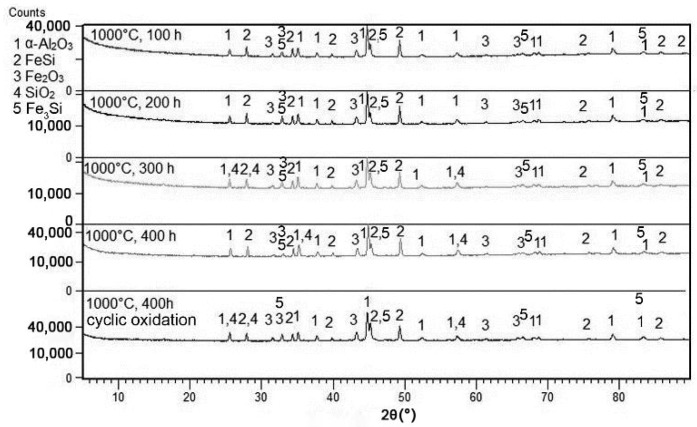
XRD patterns of FeAl20Si20 alloy after various durations of oxidation at 1000 °C in air.

Isothermal oxidation tests at 800 and 1000 °C revealed that a protective layer of aluminium oxide with minor admixtures of Fe_2_O_3_ and SiO_2_ was formed (Figure 3 and Figure 4). In addition, FeSi andFe_3_Si phases were detected, which originate from the matrix below the oxide layer. The reason why the ternary Fe–Al–Si phase was not detected is discussed below. The main difference between the oxidation at 800 and 1000 °C is in the allotropic modification of aluminium oxide, which covers the surface of the material. At 1000 °C, α-Al_2_O_3_ (corundum, trigonal) is formed. On the other hand, γ-Al_2_O_3_ (cubic) is formed at 800 °C. 

**Figure 5:**
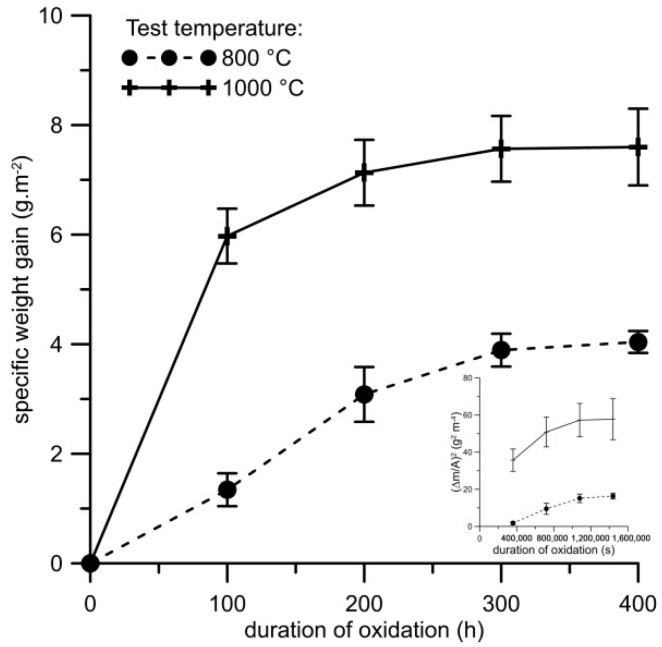
Dependence of specific weight gain (g m^−2^) on the duration of isothermal oxidation at 800 and 1000 °C in air for FeAl20Si20 alloy, with the squared value of specific weight gain vs. time as insert.

At the first stage of oxidation, a mixture of Al_2_O_3_ and Fe_2_O_3_ is formed at 1000 °C. After 300 h, the formation of SiO_2_ was detected (Figure 5). At a temperature of 800 °C, SiO_2_ was detected by XRD after only 100 h of oxidation (Figure 4).

The oxidation rate of the alloy, represented by weight gains caused by isothermal oxidation, is ca. two times lower at 800 °C than at 1000 °C (Figure 5). The growth of the oxide layer at 800 °C almost follows the parabolic law between 100 and 300 h, as seen on the insert in Figure 5, and it slows down after 300 h. This means that the oxidation is controlled by the diffusion of oxygen or aluminium through the oxide layer, and thus follows parabolic law. From the growth rate, it cannot be determined whether the layer grows inwards by oxygen diffusion or outwards by diffusion of aluminium to the surface because the diffusion rates of aluminium and oxygen in aluminium oxide are comparable [39]. The lowering of the oxidation rate at the end of the test is probably caused by the changes in chemical and phase composition below the oxide layer, as discussed below. At 1000 °C, the oxidation was much faster at the beginning, which is probably caused by reaction-controlled oxidation. After reaching a certain thickness of the oxide layer, the process changes to the diffusion-controlled mode. At the end of the oxidation test, the oxidation is slowed down due to the same effects as at 800 °C. 

**Figure 6:**
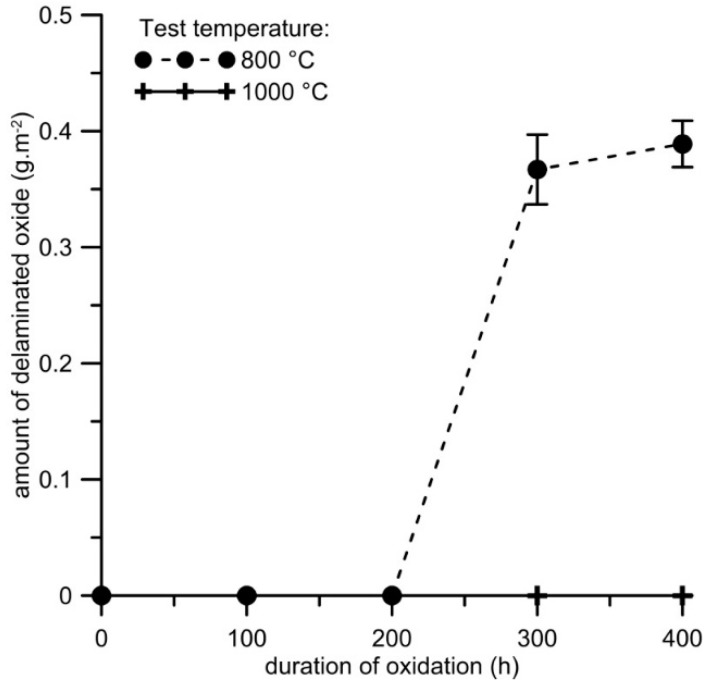
Dependence of the amount of delaminated oxide (g 006^−2^) on the duration of isothermal oxidation at 800 and 1000 °C in air for FeAl20Si20 alloy.

This shows that the oxide layer containing α-Al_2_O_3_ has superior protective effect, which was also confirmed by spallation of the oxide layer during the test. In the case of samples tested at 800 °C, the amount of delaminated oxides up to 0.4 g·m^−2^ was detected after 300–400 h of oxidation (Figure 6). On the contrary, no spallation was detected during isothermal oxidation at 1000 °C. The Pilling-Bedworth ratio [40] was calculated for this material and α-Al_2_O_3_ oxide layer as ca. 1.7, which indicates a layer with protective effect (a Pilling-Bedworth ratio below 1 indicates that no continuous oxide layer is formed on the surface, while a ratio above 2 implies layer spallation [40]).

Cyclic oxidation was also tested to prove the adherence of the oxide layer during cooling and heating up to the test temperature. 

**Figure 7:**
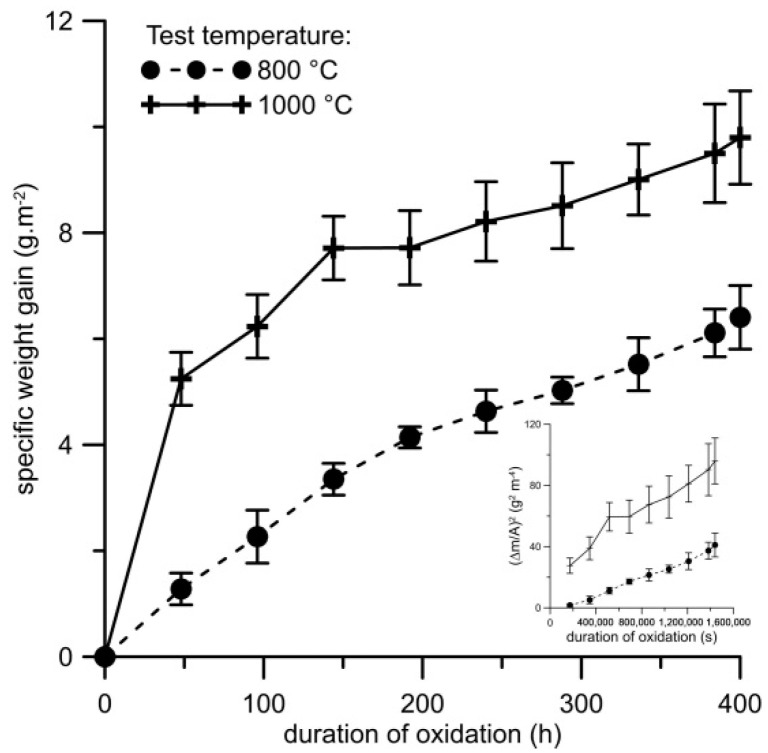
Dependence of specific weight gain (g m^2^) on duration of cyclic oxidation at 800 and 1000 °C in air for FeAl20Si20 alloy, with the squared value of specific weight gain vs. time as insert.

**Figure 8:**
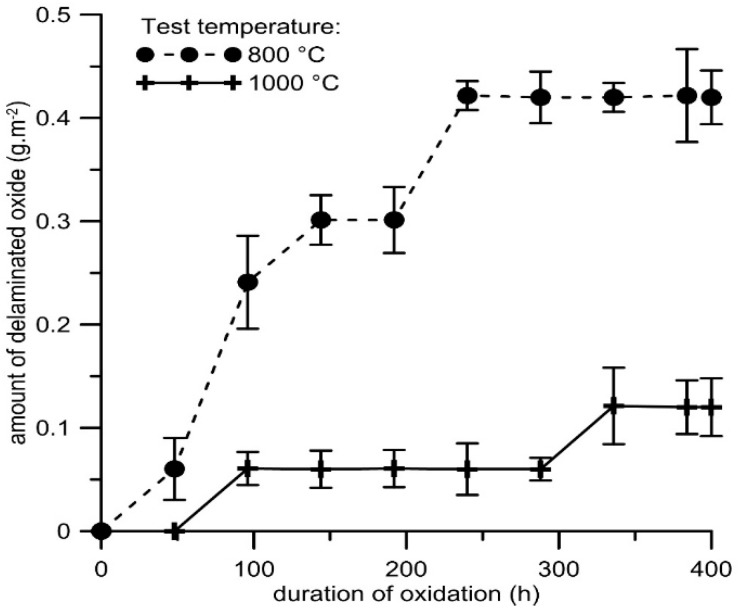
Dependence of the amount of delaminated oxide (g·m^−2^) on duration of cyclic oxidation at 800 and 1000 °C in air for FeAl20Si20 alloy.

The weight gains due to cyclic oxidation (Figure 7) were slightly higher than during the isothermal test (Figure 5). At both temperatures, the oxidation follows parabolic law with just minor deviations, as can be seen in the insert in Figure 7. The calculated parabolic rate constants are slightly higher than during the isothermal oxidation (Table 4), probably due to defects in the oxide layers caused by the stresses induced during thermal cycling. The spallation of the oxide layer was observed at both test temperatures, but more significantly at 800 °C (Figure 8). However, the amounts of delaminated oxides were very low (less than 0.2 g m^−2^ at 1000 °C and 0.5 g·m^−2^ at 800 °C). 

**Figure 9:**
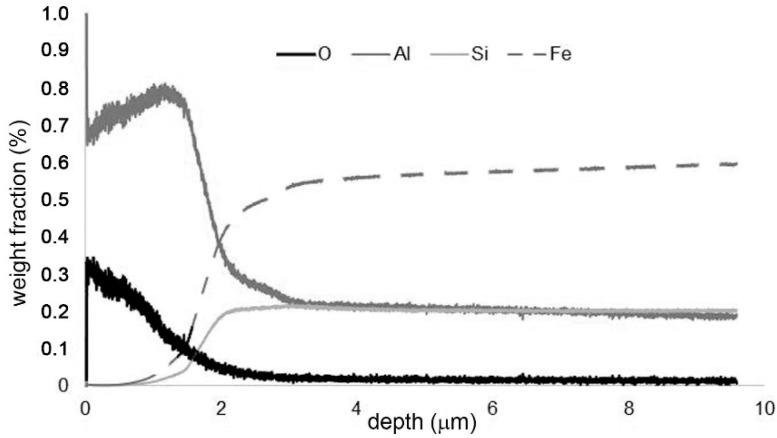
Concentration-depth profile of FeAl20Si20 alloy after cyclic oxidation at 800 °C for 400 h.

**Figure 10:**
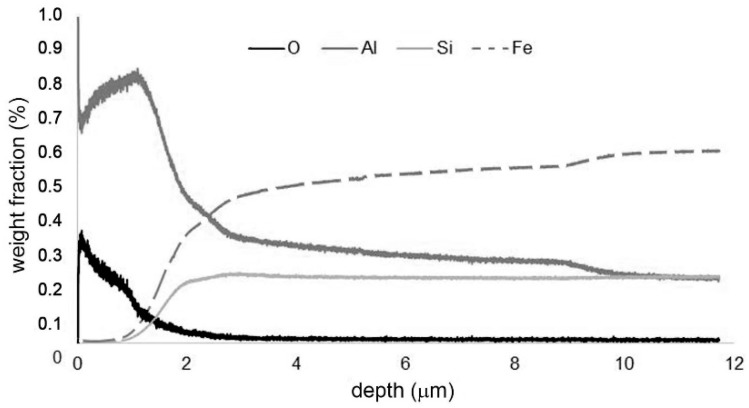
Concentration-depth profile of FeAl20Si20 alloy after cyclic oxidation at 1000 °C for 400 h.

To describe the chemical composition of the oxide layers, concentration-depth profiles of the present elements on the samples after cyclic oxidation were measured by GDOES (Figure 9 and Figure 10). The high content of aluminium in the whole surface layer confirms that aluminium oxide is the main constituent of the oxide layer. The GDOES elemental profile also shows that the material is enriched by silicon and depleted by aluminium below the oxide layer. This implies that oxidation probably proceeds on the outer surface, controlled by the diffusion of aluminium through the oxide layer.

**Figure 11:**
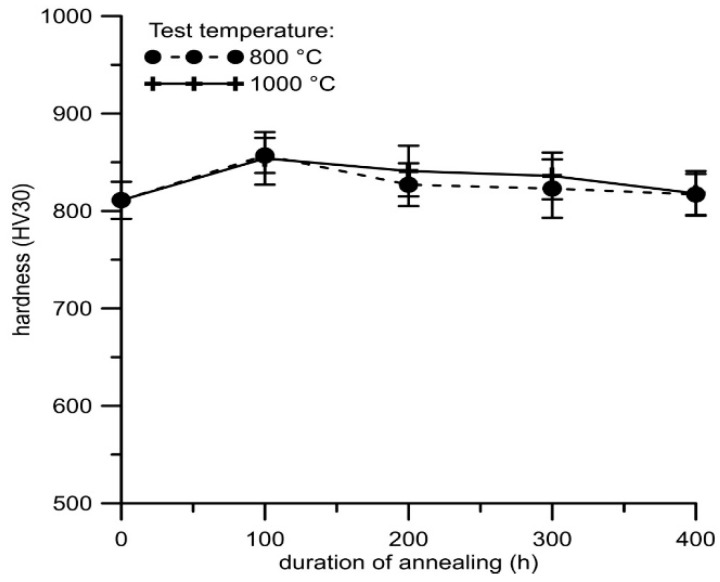
Hardness of FeAl20Si20 alloy after annealing at 800 and 1000 °C vs. duration of annealing.

Thermal stability was evaluated by the measurement of Vickers hardness (HV 30) after annealing at 800 and 1000 °C (Figure 11). The measurements were carried out in the core of the material on a crosscut. Only minor variations in the hardness can be seen after annealing at both temperatures, mostly lower than the standard deviation of the results. This indicates that the alloy is highly thermally stable, and that no significant changes in the structure occur during high-temperature exposure.

### 3.4. Corrosion in Water-Based Electrolytes

**Figure 12:**
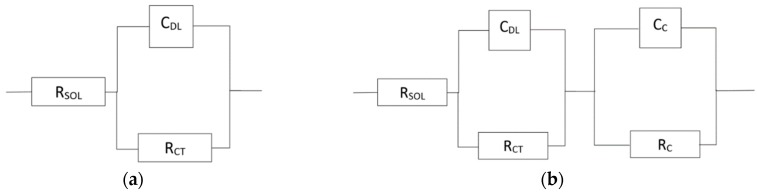
(**a**) Equivalent circuit with one RC couple (active surface), (**b**) Equivalent circuit with two RC couples (passive surface), (RSOL—resistance of the solution; RC—resistance of the passive layer; RCT—charge transfer resistance; CDL—capacitance of electrical double layer; CC—capacitance of passive layer).

The surface of samples shows two variations of behaviour in water-based environments. One is completely active, without passive layer, and is described by an equivalent circuit in Figure 12a. The second one is a surface with passive layer, as shown in Figure 12b. 

**Figure 13:**
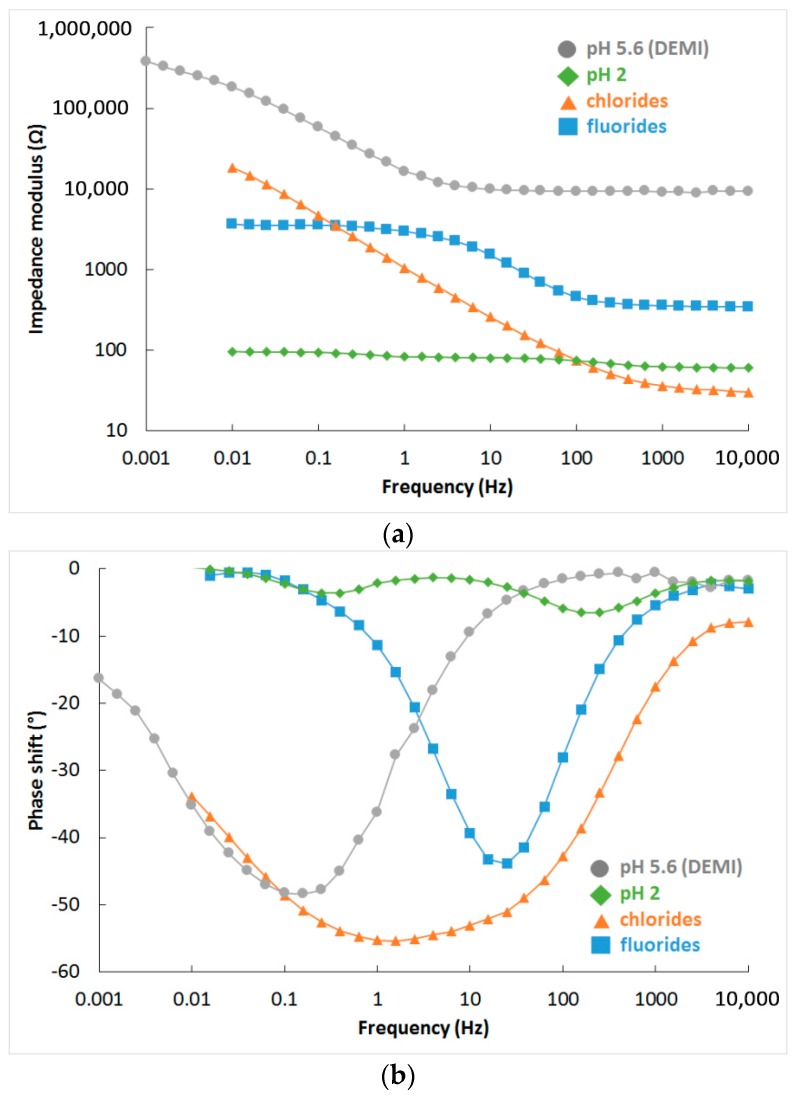
Bode plots of EIS spectra for different environments: (**a**) impedance modulus and (**b**) phase shift.

**Figure 14:**
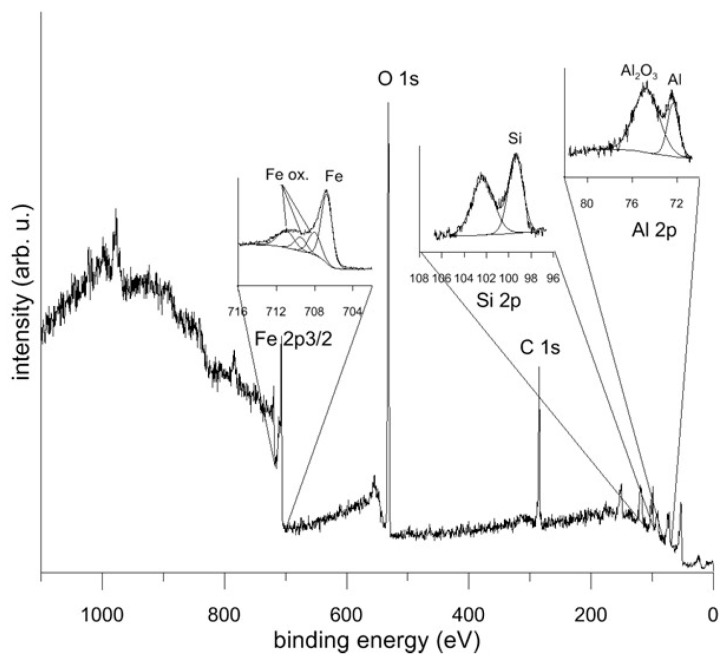
XPS spectrum of passivation layer on FeAl20Si20 formed in the electrolyte of pH = 5.6.

The corrosion behaviour of the FeAl20Si20 alloy is presented in Figure 13. Phase shift (Figure 13b) shows two time constants in spectra of demi water, chloride solution, and sulphuric acid with pH 2. Both time constants in demineralised water and chloride solution are close to each other and with high values of impedance modulus (Figure 13a). This means that there is thick natural passive layer based on aluminium oxide, as shown by XPS (Table 5, Figure 14). Low pH dissolves the aluminium oxide passive layer and it is probably replaced by a very thin passive layer based on silicon oxide. The spectra recorded in low pH solutions still show two time constants, but significantly separated, and the impedance modulus is low. The fluoride solution does not allow for the formation of any passive layer, and the surface is completely active with only one time constant in the EIS spectra (Figure 13). 

**Figure 15:**
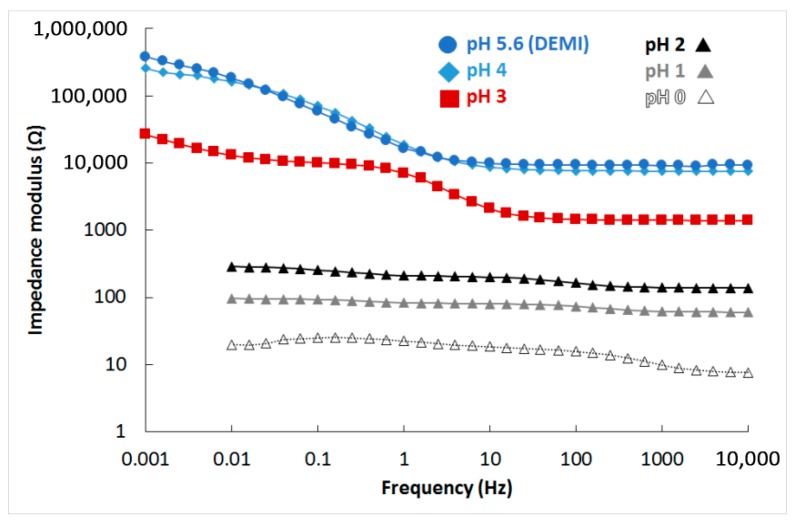
Bode plot (impedance modulus) in sulphuric acid solutions.

A summary of the results in sulphuric acid solution is given in Figure 15. The surface was covered by a thin layer of silicon oxide in the range of pH 0–2. There is a transition value of pH 3, where some aluminium oxide passive layer was present on the surface, but it has poorer quality when compared to spectrum at pH 4, which is the same as spectrum in non-aggressive demineralised water (pH 5.6). 

The complete results of spectra fitting are given in Table 6. The values of charge transfer resistance (RCT) are high for pH 3–5.6 and for chloride solution, when aluminium-based passive layer is present on the surface. Values corresponding to low pH are much lower since the silicon oxide-based passive layer has poor protective properties. The RCT is even lower than in fluorides when no passive layer is present on the surface, but material dissolution is driven by slow oxygen cathodic reduction unlike the fast hydrogen cathodic reaction in low pH environments. The passive layer resistance (RC) shows the conductivity of the layer, which is high for natural aluminium passive layer at pH 4 and 5.6 and lower for environments whereby the passive layer starts to be attacked (pH 3 and chloride solution), and it is the lowest for the silicon oxide-based passive layer. 

## 4. Discussion

The presented results for mechanical properties revealed the anomalous temperature dependence of both the yield strength and ultimate compressive strength. Among Fe–Al and Fe–Si phases, the anomalous behaviour of mechanical properties has been already reported for Fe_3_Al, FeAl, and Fe_3_Si phases [41,42,43,44,45]. However, only a weak effect has been described for Fe_3_Si, corresponding to lower temperatures [45]. In addition, the silicides formed isolated particles in the investigated alloy. This implies that the matrix—Fe_3_Al_2_Si_3_ ternary phase—probably also exhibits strong anomalous behaviour of the yield strength and ultimate tensile strength. This was not expected due to the crystal structure of this phase, which is triclinic (P-1). For definitive proof of the behaviour of this phase, samples containing pure Fe_3_Al_2_Si_3_ phase would have to be prepared and tested. 

The anomaly of YS and UCS at temperatures around 500 °C give rise to an interesting range of applications. These temperatures are common for exhaust valves of diesel internal combustion engines. During normal operation, the temperature reaches approximately 400 °C [45]. However, during the cleaning procedure of the filter of solid particles, the temperature increases to ca. 500 °C [46]. These conditions were demonstrated to be optimal for the use of this alloy. As the other results presented above show, the other characteristics (wear resistance, cyclic oxidation behaviour) also favour this material for this kind of application. 

The oxidation tests at high temperatures were consistent with the oxidation mechanism previously published for this alloy prepared by reactive sintering [12]. During oxidation, a layer of aluminium oxide is formed. The reason for the predominance of aluminium oxide is its high thermodynamic stability, as compared with iron oxide and silicon oxide (Table 7) [47]. 

At 800 °C, the oxidation product is γ-Al_2_O_3_ with cubic structure, which is then transformed to δ-Al_2_O_3_ during long-term exposure [48]. Such a transformation causes internal stresses which support the cracking and delamination of the oxide layer. On the other hand, the layer of α-Al_2_O_3_ formed at 1000 °C is stable and does not undergo any change during further exposure. Therefore, the spallation of the oxide layer is lower at 1000 °C than at 800 °C. Due to the formation of aluminium oxide, the zone below the oxide layer is depleted by aluminium. In this alloy, it leads to following transformation of the ternary phase to FeSi, which was detected below the surface (Figure 4 and Figure 5):

(4) $$Al_{2}Fe_{3}Si_{3}+\frac{3}{2}O_{2}\to Al_{2}O_{3}+3FeSi$$

Iron silicides are known to be highly oxidation-resistant [49]. Therefore, this silicon-enriched zone acts as a secondary protection when defects in the oxide layer occurs. This is probably also a reason why the oxidation slows down with oxidation duration (see the insert in Figure 5). Due to aluminium depletion, the source of aluminium decreases and is replaced by silicides. 

Silicon-enriched zones also help to stabilise the mechanical properties in the near-surface area in high-silicon Fe–Al–Si alloys, as previously shown [12]. In binary Fe–Al alloys, the softer aluminium-deficient layer of Fe_3_Al, or even Fe, can be expected. Therefore, other mechanical properties (wear resistance, fatigue life, creep limit) can also probably be negatively affected in the near-surface area in Fe–Al and Fe–Al–Si alloys with a lower amount of silicon.

A passive layer based on aluminium oxide is also responsible for exceptional corrosion resistance in electrolytes. This layer is stable at pH above 3. Below this value, an oxide film based on SiO_2_ is probably formed with a lower protective effect. Therefore, this material should be used in environments with pH values above 3. The use of fluoride solutions is not recommended because the passive layer does not form in this kind of electrolyte due to formation of water-soluble (AlF_6_)^3−^ complexes [50]. 

Based on the factors discovered above, this alloy can be one of the solutions for a problem currently being addressed by the European Commission—substitution of critical raw materials [11]. In certain applications, it could possibly substitute chromium-containing alloys (corrosion-resistant and heat-resistant steels) or chromium-, molybdenum- and tungsten-alloyed tool steels.

## 5. Conclusions

In this work, the FeAl20Si20 alloy (in wt %) was prepared by the combination of unique ultrahigh-energy mechanical alloying and spark plasma sintering. The alloy exhibited very fine structure, composed of silicides (Fe_3_Si and FeSi) and ternary phase (Fe_3_Al_2_Si_3_). At room temperature, the ultimate compressive strength of the alloy was nearly 1100 MPa, while it increased to ca. 1500 MPa at 500 °C. The applied technology enabled achievement of a fracture toughness of 3.50 ± 0.33 MPa·m^1/2^ at room temperature, which is almost ten times higher than the value determined for the material of the same composition prepared by casting. This value is almost comparable to ceramic materials. The abrasive wear rate of the alloy is a lower than in the case of the heat-treated AISI D2 cold work tool steel, which is considered as highly wear-resistant tool material. The FeAl20Si20 alloy exhibits excellent oxidation resistance at 800 and 1000 °C in air. From this viewpoint, the performance of the material at 1000 °C is better than at 800 °C, due to the formation of a highly protective α-Al_2_O_3_ layer. In water-based electrolytes, the material behaves passively at pH values above 3. The passivity is a result of the presence of an aluminium oxide layer on the surface. Under this pH value, the aluminium oxide layer dissolves and it is replaced by silicon oxide, which has a lower protective effect. Due to its superior chemical resistance, wear resistance, and mechanical behaviour, the material could be applicable in the automotive industry for the manufacture of exhaust valves, wear parts, or as a material for aggressive environments.

## Figures and Tables

**Table 1:** Average chemical composition of individual phases in FeAl20Si20 alloy (determined by EDS).

Phase	Chemical Composition (wt %)		
	Fe	Al	Si
Fe_3_Si	83.9 ± 0.6	7.2 ± 0.6	8.9 ± 0.5
FeSi	65.5 ± 1.4	12.1 ± 3.2	22.4 ± 3.4
Fe_3_Al_2_Si_3_	46.0 ± 4.3	27.6 ± 4.2	27.4 ± 4.3

**Table 2:** Weight fraction, lattice parameters, and crystallite size of individual phases in FeAl20Si20 alloy (determined by Rietveld refinement of XRD pattern).

Phase	Weight Fraction (wt %)	Space Group	Lattice Parameters (× 10^−10^ m)	Crystallite Size (nm)
Fe_3_Si	33.0 ± 0.4	Fm3m	5.6934 ± 0.0004	23 ± 1
FeSi	37.5 ± 0.4	P2_1_3	4.5337 ± 0.0001	45 ± 1
Fe_3_Al_2_Si_3_	29.5 ± 0.4	P-1	4.6512 ± 0.0001 6.3261 ± 0.0002 7.4990 ± 0.0002	39 ± 1

**Table 3:** Mechanical properties (yield strength and ultimate compressive strength) of FeAl20Si20 alloy in compression.

Temperature (°C)	Yield Strength (MPa)	UCS (MPa)
25	1071 ± 110	1085 ± 115
400	1101 ± 129	1140 ± 138
500	1163 ± 78	1508 ± 239
600	597 ± 103	1079 ± 236
700	348 ± 7	644 ± 1

**Table 4:** Parabolic rate constants (*k_p_*) of isothermal and cyclic oxidation at 800 and 1000 °C.

Test Mode	Cyclic Oxidation Test		Isothermal Oxidation Test	
**Temperature (°C)**	**800 °C**	**1000 °C**	**800 °C**	**1000 °C**
***k_p_* (g^2^·m^−4^·s^−1^)**	2.58 × 10^−5^	7.22 × 10^−5^	1.09 × 10^−5^	5.46 × 10^−5^

**Table 5:** Chemical composition of passivation layer on FeAl20Si20 formed in the electrolyte of pH = 5.6.

Element	Fe	Al-Metallic	Al-Oxide	Si	C	O
Concentration (atom %)	3.0	5.9	15.8	17.1	29.2	29.0

**Table 6:** Results of EIS spectra fitting (RSOL—resistance of the solution; RC—resistance of the passive layer; RCT—charge transfer resistance; CDL—capacitance of electrical double layer; CC—capacitance of passive layer).

Parameter	pH 0	pH 1	pH 2	pH 3	pH 4	pH 5.6
R_SOL_ (Ω·m^2^)	5.91 × 10^−4^	4.88 × 10^−3^	1.10 × 10^−2^	1.11 × 10^−1^	6.03 × 10^−1^	7.36 × 10^−1^
R_CT_ (Ω·m^2^)	7.76 × 10^−4^	1.19 × 10^−3^	7.92 × 10^−3^	6.28	9.54	23.54
R_C_ (Ω·m^2^)	7.83 × 10^−4^	1.57 × 10^−3^	5.40 × 10^−3^	5.88 × 10^−1^	9.19	9.36
C_DL_ (S·s^α^·m^−2^)	3.99 × 10^2^	4.25 × 10^2^	2.05 × 10^2^	8.97	1.24	9.67 × 10^−1^
α_DL_	0.64	0.85	0.77	0.53	0.77	0.77
C_C_ (S·s^α^·m^−2^)	2.28	1.21	1.22	2.42 × 10^−1^	2.42 × 10^−1^	4.17 × 10^−1^
α_C_	0.82	0.90	0.84	0.94	0.78	0.74

**Table 7:** Gibbs energy (ΔG_f_) of formation of oxides at 800 °C (calculated on the basis of published data [47]).

Oxide Formula	ΔG_f_ (800 °C) (kJ·mol^−1^)
Al_2_O_3_	−1778
Fe_2_O_3_	−982
SiO_2_	−983

## References

[1] Zhu X., Yao Z., Gu X., Cong W., Zhang P. (2009). Microstructure and corrosion resistance of Fe-Al intermetallic coating on 45 steel synthesized by double glow plasma surface alloying technology. Trans. Nonferrous Met. Soc. China.

[2] Borsig A. (1894). Zusatz von Aluminium zum Roheisen. Stahl Eisen.

[3] Kratochvíl P. (2008). The history of the search and use of heat resistant Pyroferal alloys based on FeAl. Intermetallics.

[4] Zamanzade M., Vehoff H., Barnoush A. (2013). Effect of chromium on elastic and plastic deformation of Fe3Al intermetallics. Intermetallics.

[5] Ferreira P.I., Couto A.A., de Paola J.C.C. (1995). The effects of chromium addition and heat treatment on the microstructure and tensile properties of Fe-24Al (at.%). Mater. Sci. Eng. A.

[6] Balasubramaniam R. (1996). On the role of chromium in minimizing room temperature hydrogen embrittlement in iron aluminides. Scr. Mater..

[7] Zhang Z., Sun Y., Guo J. (1995). Effect of niobium addition on the mechanical properties of Fe3Al-based alloys. Scr. Metall. Mater..

[8] Janda D., Fietzek H., Galetz M., Heilmaier M. (2013). The effect of micro-alloying with Zr and Nb on the oxidation behavior of Fe3Al and FeAl alloys. Intermetallics.

[9] Sundar R.S., Deevi S.C. (2003). High-temperature strength and creep resistance of FeAl. Mater. Sci. Eng. A.

[10] Klein O., Baker I. (1992). Effect of chromium on the environmental sensitivity of FeAl at room temperature. Scr. Metall. Mater..

[11] Grilli M.L., Bellezze T., Gamsjager E., Rinaldi A., Novak P., Balos S., Piticescu R.R., Ruello M.L. (2017). Solutions for Critical Raw Materials under Extreme Conditions: A Review. Materials (Basel).

[12] Novák P., Zelinková M., Šerák J., Michalcová A., Novák M., Vojtěch D. (2011). Oxidation resistance of SHS Fe-Al-Si alloys at 800 °C in air. Intermetallics.

[13] Li H., Zhang J., Young D.J. (2012). Oxidation of Fe–Si, Fe–Al and Fe–Si–Al alloys in CO_2_–H_2_O gas at 800 °C. Corros. Sci..

[14] Kratochvíl P., Schindler I. (2007). Hot rolling of iron aluminide Fe28.4Al4.1Cr0.02Ce (at%). Intermetallics.

[15] Schindler I., Kratochvíl P., Prokopčáková P., Kozelský P. (2010). Forming of cast Fe—45 at.% Al alloy with high content of carbon. Intermetallics.

[16] Šíma V., Kratochvíl P., Kozelský P., Schindler I., Hána P. (2009). FeAl-based intermetallics cast in an ultrsonic field. Int. J. Mater. Res..

[17] Novák P., Michalcová A., Voděrová M., Šíma M., Šerak J., Vojtěch D., Wienerová K. (2010). Effect of reactive sintering conditions on microstructure of Fe–Al–Si alloys. J. Alloy Compd..

[18] Xia Y., Li L., Li L. (2014). Effect of grain refinement on fracture toughness and fracture mechanism in AZ31 magnesium alloy. Procedia Mater. Sci..

[19] Schwarz K.T., Kormout K.S., Pippan R., Hohenwarter A. (2017). Impact of severe plastic deformation on microstructure and fracture toughness evolution of a duplex-steel. Mater. Sci. Eng. A.

[20] Vaidya M., Prasad A., Parakh A., Murty B.S. (2017). Influence of sequence of elemental addition on phase evolution in nanocrystalline AlCoCrFeNi: Novel approach to alloy synthesis using mechanical alloying. Mater. Des..

[21] Fang Q., Kang Z., Gan Y., Long Y. (2015). Microstructures and mechanical properties of spark plasma sintered Cu–Cr composites prepared by mechanical milling and alloying. Mater. Des..

[22] Froes F.H., Suryanarayan C., Russell K., Li C.-G. (1995). Synthesis of Intermetallics by Mechanical Alloying. Mater. Sci. Eng. A.

[23] Al-Joubori A., Suryanarayana C. (2015). Synthesis of metastable NiGe2 by mechanical alloying. Mater. Des..

[24] Naghiha H., Movahedi B., Asadabad M.A., Mournani M.T. (2017). Amorphization and nanocrystalline Nb3Al intermetallic formation during mechanical alloying and subsequent annealing. Adv. Powder Technol..

[25] Molladavoudi A., Amirkhanlou S., Shamanian M., Ashrafizadeh F. (2012). Synthesis and characterization of nanocrystalline CoTi intermetallic compound prepared by mechanical alloying. Mater. Lett..

[26] Novák P., Kubatík T., Vystrčil J., Hendrych R., Kříž J., Mlynár J., Vojtěch D. (2014). Powder metallurgy preparation of Al-Cu-Fe quasicrystals using mechanical alloying and Spark Plasma Sintering. Intermetallics.

[27] Chuvildeev V.N., Panov D.V., Boldin M.S., Nokhrin A.V., Blagoveshchensky Y.V., Sakharov N.V., Shotin S.V., Kotkov D.N. (2015). Structure and properties of advanced materials obtained by Spark Plasma Sintering. Acta Astronaut..

[28] Zhang Z., Liu Z., Lu J., Shen X., Wang F., Wang Y. (2014). The sintering mechanism in spark plasma sintering–Proof of the occurrence of spark discharge. Scr. Mater..

[29] Marder R., Estourne C., Chevallier G., Chaim R. (2014). Plasma in spark plasma sintering of ceramic particle compacts. Scr. Mater..

[30] Liu J., Liang C. (2017). Microstructure characterization and mechanical properties of bulk nanocrystalline aluminium prepared by SPS and followed by hightemperature extruded techniques. Mater. Lett..

[31] Peters C.T. (1979). The relationship between Palmqvist indentation toughness and bulk fracture toughness for some WC-Co cemented carbides. J. Mater. Sci..

[32] Novák P., Vojtěch D., Šerák J. (2006). Wear and corrosion resistance of a plasma-nitrided PM tool steel alloyed with niobium. Surf. Coat. Technol..

[33] Mrowec S., Stoklosa A. (1974). Calculations of Parabolic Rate Constants for Metal Oxidation. Oxid. Met..

[34] (2008). NIST X-ray Photoelectron Spectroscopy Database.

[35] Hightower A., Fultz B., Bowman R.C. (1997). Mechanical alloying of Fe and Mg. J. Alloy Compd..

[36] Novák P., Průša F., Nová K., Bernatiková A., Salvetr P., Kopeček J., Haušild P. (2018). Application of mechanical alloying in synthesis of intermetallics. Acta Phys. Pol. A.

[37] de Farias Azevedo C.R., Flower H.M. (2000). Calculated ternary diagram of Ti–Al–Si system. Mater. Sci. Technol..

[38] Farzin-Nia F., Sterrett T., Sirney R. (1990). Effect of machining on fracture toughness of corundum. J. Mater. Sci..

[39] Heuer A.H. (2008). Oxygen and aluminum diffusion in α-Al2O3: How much do we really understand?. Eur. Ceram. Soc..

[40] Proff C., Abolhassani S., Lemaignan C. (2013). Oxidation behaviour of zirconium alloys and their precipitates—A mechanistic study. J. Nucl. Mater..

[41] Morris D.G., Munoz-Morris M.A. (2010). A re-examination of the pinning mechanisms responsible for the stress anomaly in FeAl intermetallics. Intermetallics.

[42] George E.P., Baker I. (1998). Thermal vacancies and the yield anomaly of FeAl. Intermetallics.

[43] Morris D.G., Munoz-Morris M.A. (2005). The stress anomaly in FeAl–Fe3Al alloys. Intermetallics.

[44] Brinck A., Neuhäuser H. (2004). Yield stress and dislocation mechanisms in the D03 ordered intermetallic phase Fe3Al in the temperature range 240–500K. Mater. Sci. Eng. A.

[45] Schaefer H.-E., Frenner K., Wurschum R. (1999). High-temperature atomic defect properties and diffusion processes in intermetallic compounds. Intermetallics.

[46] Castellano J., Chaudhari A., Bromham J. (2013). Adaptive Temperature Control for Diesel Particulate Filter Regeneration. SAE Tech. Pap..

[47] Barin I. (1995). Thermochemical Data of Pure Substances.

[48] Souza Santos H., Souza Santos P. (1992). Pseudomorphic formation of aluminas from fibrillar pseudoboehmite. Mater. Lett..

[49] Roy S.K., Fasasi A., Pons M., Galerie A., Caillet M. (1993). High-temperature oxidation behaviour of laser-surface alloyed iron-silicon coatings on iron. J. Phys. IV.

[50] Corbillon M.S., Olazabal M.A., Madariaga J.M. (2008). Potentiometric Study of Aluminium-Fluoride Complexation Equilibria and Definition of the Thermodynamic Model. J. Solut. Chem..

